# How to play the role of fiscal subsidies? Based on the research of photovoltaic industry in China——Based on SBM-DEA and FGLS methods

**DOI:** 10.1016/j.heliyon.2024.e32980

**Published:** 2024-06-13

**Authors:** Jing Li

**Affiliations:** XuChang university, XuChang, 461000, China

**Keywords:** Industrial development, PV industry, SBM-DEA, Fiscal compensation

## Abstract

Fiscal compensation may play either an incentive or a crowding-out role in the different enterprises of the PV industry. First, a model for evaluating the efficiency of fiscal compensation was designed. Then, an empirical analysis of the influencing factors using a panel data model was made. Results showed that fiscal compensation had an incentive effect on 73.3 % of enterprises, but it had a crowding-out effect on the remaining enterprises. The average efficiency of fiscal compensation for enterprises in the PV industry is 1.117. The average efficiency of fiscal compensation for the front-end and back-end enterprises was 1.002 and 1.231, respectively. The impact of fiscal compensation on China's photovoltaic industry has shown a downward trend over time, and the role has also changed from an incentive effect to a crowding-out effect. The size of the enterprise and the intensity of fiscal compensation will affect the efficiency of fiscal compensation. There is an inverted U-shaped relationship between the intensity of fiscal compensation in China's photovoltaic industry and the efficiency of fiscal compensation. The larger the enterprise, the more efficient the fiscal compensation will be.

## 1 Introduction

The contradiction between the infinity of energy demand and limited energy resources is one of the most important challenges to sustainable development [1]. Because of its cleanliness, richness, permanence, balance, and renewability, solar energy has become the most ideal alternative to traditional energy [2,3]. The development of solar energy is of great significance to the world's sustainable development, mitigating climate change, and environmental protection [4,5]. The use of renewable energy such as solar energy as a substitute for traditional energy can not only fundamentally solve the serious problem of ecological environment pollution and the increasing depletion of traditional energy, but also create new economic growth chances and promote the progress of human society [6,7].

Solar energy generally refers to the radiation energy of sunlight, which is mainly utilized in three ways: photothermal conversion, photoelectric conversion, and photochemical conversion [[8], [9], [10]]. From the perspective of practical experience, the photovoltaic industry (PV) that realizes power generation through photoelectric conversion can make better use of the characteristics of balanced distribution of solar energy, which is more in line with the requirements of future human society for energy-independent and decentralized production [11,12]. According to the forecast of the European Commission's Joint Research Centre, solar power will play a vital role in the world's power supply by 2030, reaching more than 10 %; solar power will account for 20 % of the total energy consumption in 2050 and will play a leading role in the energy structure by the end of this century. From the perspective of the PV industry itself, the industrial chain is long, involving many key industries of the national economy, such as mineral, chemical, metallurgical, semiconductor, construction, real estate, electric power, and so on. The potential of the terminal application market is huge. Because the PV industry has the characteristics of strategic characteristics, namely technology-intensive, and capital-intensive, among many others, many countries regard the application and industrialization of PV technology as their key development undertakings. To promote the development of the PV industry, governments have formulated and implemented many industrial support policies [[13], [14], [15]].

Since 2006, China's PV industry has had a strong momentum of development. However, since the United States launched an anti-dumping investigation into Chinese PV products (including solar cells and solar panels) in 2011, Chinese PV exports have fallen sharply, and many PV companies have been damaged. Between 2011 and 2012, exports fell by 42 %. There are many reasons why the United States carries out anti-dumping investigations on Chinese PV products, such as vicious low-price competition among Chinese PV enterprises, the ineffective response of Chinese PV enterprises, overproduction of PV products. But the more essential reason is that there are problems in China's PV industrial policy and fiscal policy [16,17]. Even with the United States' containment, China's PV industry is still developing rapidly. Under the "double carbon" goal, China has accelerated the development of renewable energy power, especially the PV industry. To encourage the development of the PV industry, the Chinese government began to implement electricity price subsidies for photovoltaic power in 2013. However, the long-term subsidies to the photovoltaic industry have reduced the efficiency of subsidies, and the subsidy amount and subsidy gap have continued to increase. From 2018, the Chinese government began to reduce the subsidy standard for PV generation. In 2021, the subsidy for industrial and commercial users was canceled [18]. From practical experience, the PV industry is a policy-driven industry [19,20]. One of the reasons for this is the high cost caused by the low level of technological development. At present, the cost of solar PV power generation is about 10–20 times that of coal-fired. Therefore, many countries have given strong support to their PV industry through inclined policies, such as the Asahi Seven-year Plan of Japan, the Thousand Subscriptions Plan of Germany, Million Roof Plan of the United States [21]. As a result, the PV industry has gained opportunities for rapid development. In 2010, China listed the renewable energy industry represented by PV as a strategic emerging industry, which means that the PV industry has become the key support object of the Chinese government's industrial policy. In particular, the Chinese government promulgated interim measures for the Management of Special Funds for the Development of Strategic Emerging Industries in 2012, indicating that the Chinese government has gradually focused its policies on incentives such as fiscal compensation. The government's huge fiscal compensation to the PV industry gives China's PV industry a distinctive feature of fiscal compensation. Taking this opportunity, China's PV industry has made great progress in a short period, and the installed capacity of PV is growing continuously.

However, the adjustment of consumption patterns in the United States and other developed countries has caused a great structural impact on China's export-oriented manufacturing industry in the context of the post-fiscal crisis [16]. At the same time, European countries, the United States, and other countries put forward the "re-industrialization" strategy including vigorously developing biology, information, new energy, and other emerging industries, trying to revitalize the manufacturing industry. The newly industrializing countries and regions such as ASEAN, India, and Latin America are also speeding up the adjustment of industrial structure and promoting industrial upgrading [11]. Besides, due to the rapid rise in the cost of resources and energy, labor, land, and other factors, China's manufacturing industry is gradually losing its low-price competitive comparative advantage brought about by low factor costs. The PV industry is a typical representative of the challenges and opportunities faced by China's manufacturing industry in the complex internal and external environment, and its development process also presents problems such as both sides (technology and marketing) outside, and structural overcapacity, among others. These problems have aroused widespread discussion on China's industrial policy [16].

Industrial policy (mainly fiscal compensation) has two sides, and there is no agreement on this issue in the academic community. on the one hand, fiscal compensation has an incentive effect on industrial development, such as encouraging enterprises to increase R&D investment [22], promoting export and domestic value-added rates, increasing innovative output and financing opportunities [23,24], improving production efficiency [25,26], promoting green innovation [27], among many others. On the other hand, fiscal compensation has a crowding-out effect on industrial development, such as increasing the purchase cost of enterprises [28], reducing the utilization rate of production capacity [29], reducing investment efficiency [30], increasing cost stickiness [31], crowding-out innovation performance and quality of enterprises [32].

Furthermore, fiscal efficiency is an important metric to measure the fiscal effect. The existing research is mainly focused on the calculation of the fiscal expenditure efficiency of government departments [33,34]. At the same time, it also involves research on the efficiency of the government's special fiscal expenditure in public fields, such as agriculture, environmental protection, public transport, and scientific and technological innovation [35]. In the field of industrial development, Li et al. used fiscal compensation funds, government procurement and other indicators as input variables to measure the efficiency of Guangdong Province's fiscal compensation for the development of strategic emerging industries [36]. The efficiency measurement method used in the existing literature is mainly the frontier analysis, especially the Data Envelopment Analysis (DEA), which has been widely used because of its support for multi-input and multi-output.

Therefore, the existing research has laid a theoretical and methodological foundation for evaluating the effect of fiscal compensation on the sustainable development of the PV industry. However, there are the following limitations: in the first place, the existing research on the effect of PV industry policy is mostly focused on tax policy, export policy, and government reserve policy, while the research on the effect of fiscal compensation is still scant, and further investigation is required. Secondly, most of the existing studies have discussed the effects of fiscal compensation on industrial development in a certain field, such as industrial R&D, export, investment, and financing, but failed to comprehensively examine the all-round effects of fiscal compensation. Industrial upgrading is the most important development goal of the PV industry. The indicator of total factor productivity, which is the core indicator for comprehensively measuring the level of industrial sustainable development and upgrading, has not been investigated in the literature on the effect of fiscal compensation [37]. Thirdly, efficiency measurement methods are mostly applied to the study of the effect of government public fiscal expenditure, and few pieces of literature measure the effect of government fiscal compensation on industrial sustainable development from the perspective of efficiency. Last, the existing studies are mostly analyzed under the framework of single-factor productivity, and the calculation model of fiscal compensation efficiency needs to be improved [38].

As one of the strategic emerging industries in China, why is the PV industry also experiencing overcapacity that is frequently only found in traditional industries such as steel and electrolytic lead? Does the PV industry inevitably fall into the strange circle of *policy support-overcapacity-ineffective* regulation? How can the PV manufacturing industry change from big to strong? How should China's industrial policy be improved to achieve the goal of promoting the sustainable development of the PV industry?

Based on this, we theoretically analyze the inherent logic of fiscal compensation on the development of China's PV industry, put forward corresponding research hypotheses, and explore the possible differences and main influencing factors of fiscal compensation effects in different stages of the industry chain. In the empirical aspect, based on the data of Chinese PV-listed companies from 2010 to 2019, fiscal compensation is introduced into the total factor productivity analysis framework of the PV industry as an external environmental variable. Considering the role of industry input and external fiscal input, a fiscal compensation efficiency evaluation model based on the SBM-DEA four-stage analysis method is constructed. We judge the fiscal compensation effect of PV industry sustainable development from the perspective of *incentive or crowding-out* effect and distinguish the difference between the front end and back end of the PV industry chain to test the fiscal compensation effect. At the same time, from the perspective of enterprise characteristics, we construct an analysis model of the factors affecting the efficiency of fiscal compensation, examine the main factors affecting the fiscal compensation effect of the development of the PV industry, and put forward suggestions that are beneficial to policy optimization and enterprise development.

The remainder of this paper is structured as follows. The next section will discuss our theoretical basis, literature review, and hypotheses. Section 3 describes the methodology and data sources. Section 4 explains and discusses the empirical results obtained. Section 5 and Section 6 give conclusions and suggestions respectively.

## 2 Theoretical background analysis and hypotheses

Market failure theory and government intervention theory believe that the market mechanism cannot effectively allocate resources in certain circumstances, resulting in inefficient resource allocation. At this time, government involvement and intervention become necessary. However, based on the theory of industrial development and life cycle, industries and enterprises face different challenges and demands at different stages of development. As a policy tool, fiscal support has different impacts on industries and enterprises at different stages of development, and even has a crowding-out effect [39,40]. Therefore, for different parts of the PV industry chain, namely the front-end (such as mining, primary processing) and the back-end (such as deep processing, application and R&D), because of their significant attribute differences in technology complexity, capital intensity, environmental impact and market maturity, the role and form of fiscal compensation will also be different.

The front end of the PV industry chain is a traditional resource-based industry, such as the smelting of metallic silicon, which has been developed for a long time and has formed a large capacity scale and even overcapacity [41]. Overcapacity means that the supply of products in the market exceeds demand. In order to compete for limited market share, enterprises may fall into a price war, which will reduce profit margins and even be eliminated. The government provides fiscal subsidies to the front-end enterprises of the PV industry chain, which ostensibly maintains the survival and operation of the enterprises, but it distorts the factor market price, enterprise cost structure, and supply curve [40]. Under the circumstances of cost externalization and risk externalization, enterprises are prone to receive wrong signals and continue to over-invest in inefficient projects, thereby aggravating the overcapacity of the PV industry, making production resources not be allocated optimally and ultimately leading to the reduction of total factor productivity [42].

As an emerging strategic high-tech industry, the back end of the photovoltaic industry chain mainly focuses on the R & D and production of deep-processed products such as new photovoltaic materials. Due to the high technology threshold, many enterprises are in the start-up stage and are committed to technology research and development and market development. Resource-Based View believes that the competitive advantage of enterprises comes from their unique resources and capabilities, which are difficult to imitate and replace. The key to the growth and success of enterprises lies in the acquisition and utilization of these special heterogeneous resources. By meeting the capital needs of enterprises in technological research and development and capacity expansion, fiscal compensation can effectively enhance the relative competitiveness of enterprises, reduce costs and improve market competitiveness. At the same time, fiscal compensation promotes technological innovation and market development, and promotes the formation of industrial clusters and the development of regional economy. In addition, fiscal compensation represents the government's recognition of the supported PV enterprises, which can serve as an incentive for the production and innovation benefits of these enterprises [43].

Based on the above analysis, we propose the following hypotheses.

H1 Fiscal compensation has a crowding effect on the front end of the PV industry chain.

H2 Fiscal compensation has an incentive effect on the back end of the PV industry chain.

There is often an inverted U-shaped relationship between the intensity of fiscal compensation and the efficiency of fiscal compensation [40]. The investment production and technical research and development of photovoltaic enterprises have the characteristics of high cost, high risk and long cycle, which bring great challenges to the enterprises [44]. Through providing appropriate fiscal compensation, sharing risks, guiding policies and supporting research and development, the government's fiscal compensation policy can effectively alleviate these challenges, promote the growth and technological innovation of photovoltaic enterprises, and promote the sustained and healthy development of the photovoltaic industry [45]. Fiscal compensation plays an important role in promoting the investment and R & D of rare earth enterprises, but when the support is too strong, it will lead to "subsidy-seeking" behavior, reduce the enthusiasm of independent innovation and investment, and thus restrain their scale growth and technological progress. Only by reasonably designing and implementing fiscal compensation policies, encouraging enterprises to innovate independently and improving the supervision mechanism, can we effectively improve total factor productivity and achieve long-term sustainable development of enterprises.

Also, as a comprehensive reflection of the hard power of supporting facilities and organizational capital soft power, enterprise size has an important impact on the effect of fiscal compensation for industrial development. Because of their advantages in resources, capital, management ability and market influence, large enterprises can make full and efficient use of fiscal compensation to achieve more significant development effects. For the capital-and technology-intensive photovoltaic industry, the same amount of fiscal compensation funds invested in large-scale enterprises is more conducive to mobilize internal and external resources, higher project success rate and rate of return, and lower risk of fiscal compensation. The scale effect and experience effect of large-scale enterprises enable them to make more efficient use of fiscal compensation to promote technological innovation and industrial development [46]. Therefore, the larger the scale of PV enterprises, the better the fiscal compensation effect.

Based on the above analysis, we put forward the following assumptions.

H3 the relationship between the intensity of fiscal compensation and the efficiency of the fiscal compensation of the PV industry is inverted U-shaped.

H4 the scale of enterprises has a positive impact on the efficiency of the fiscal compensation of the PV industry.

## 3 Data and methods

### 3.1 Sample selection

We chose PV-listed companies as research samples from a micro perspective. According to the stock sector of PV released by iwencai.com, 40 listed companies were selected initially, and the following two types of enterprises that do not meet the research requirements were excluded. The first category is only related to PV equity participation or PV-related transactions, and PV is not its main business or not representative of the industry. The second category is those enterprises that have a short time to market or a short time to operate PV businesses whose data is not representative. Finally, a total of 30 sample enterprises are obtained. At the same time, we divide the selected sample listed enterprises into the front- and back-end of the PV industrial chain. The PV-listed companies mainly engaged in PV raw materials are included in the front-end, and the PV-listed companies mainly engaged in PV functional materials and high-end applications are included in the back-end; among the samples, 8 listed companies are included in the front-end of the PV industry chain, and 22 listed companies are included in the back-end of the PV industry chain. Based on the availability of research data, data from 2010 to 2019 were selected for analysis.

### 3.2 Model design

#### 3.2.1 Fiscal compensation efficiency measurement model

Fiscal compensation is the government's macro-control measure for the PV industry, which affects the development environment of enterprises, but is not a direct investment by the enterprises themselves. Classic DEA models, including the constant returns to scale (CCR) model and the variable returns to scale (BCC) model, are appropriate for scenarios where inputs and outputs are directly linked but are not designed to account for external environmental factors. The multi-stage DEA model is suitable for solving external environmental problems. Drawing on the practice of Wang et al. [47] and Dong et al. [40], we select the four-stage DEA model proposed by Fried et al. [48] and the efficiency measurement model, slacks-based measure (SBM) proposed by Tone [49], and establish a four-stage model based on SBM-DEA. The SBM model solves the problem that the radial model neglects the relaxation variable when measuring the inefficiency well. Based on the framework for measuring the total factor productivity of the PV industry, fiscal compensation is introduced as the environmental variable to evaluate the comprehensive efficiency and management efficiency firstly which are used to access the fiscal compensation efficiency secondly.

The basic steps of calculating the comprehensive efficiency and management efficiency of the PV industry, drawing on Dong et al. [40] research methods, are as follows.

Step 1 the SBM analysis based on the original input-output. The SBM efficiency evaluation model is employed to calculate the comprehensive efficiency of PV industry development, and the efficiency value of each decision-making unit (DMU) and the relaxation variable value of each input and output index are obtained.

$$\text{MinC}E_{\text{PV}}=\frac{1‐\frac{1}{m}\sum_{i=1}^{m}\frac{s_{i}^{‐}}{x_{\text{id}}}}{1+\frac{1}{n}\sum_{l=1}^{n}\frac{s_{i}^{+}}{y_{\text{ld}}}}$$

s.t.

(1) $$\sum_{j=1,\neq d}^{k}x_{\text{ij}}\lambda_{j}+s^{‐}=x_{d},i=1,2,\ldots ,m\;\sum_{j=1,\neq d}^{k}x_{\text{ij}}\lambda_{j}+s^{‐}=x_{d},i=1,2,\ldots ,m$$

$$j=1,2,\ldots ,k\left(j\neq d\right)\;\lambda \geq 0,s^{+}\geq 0,s^{‐}\geq 0$$

Where Comprehensive Efficiency of the PV Industry（$CE_{PV}$）is a measure of the overall efficiency of the PV industry, that is, the total factor productivity of the PV industry, reflecting the development and upgrading level of the PV industry; m and n represent the number of input factors and output factors, respectively; $x_{id}$ is the i th component in the input vector $x_{d}$ of the d th DMU, and $y_{ld}$ is the l th component in the output vector $y_{d}$ of the d th DMU; $s^{-}$ and $s^{+}$ are slack variables of the i th input factor and r th output factor, respectively; k is the number of DMU; and $y_{lj}$ are the i th input factor and the l th output factor of the j th DMU, respectively; $\lambda_{j}$ is the weight vector of the j th DMU.

Step 2 the environmental variables impact analysis based on the Tobit model. The indicators of fiscal compensation are set as environmental variables, and multiple regression equations are established with input slack as a dependent variable and environment variable as an independent variable:

(2) $$s_{\text{ij}}^{‐}=f\left(W_{\text{ij}},\alpha_{i},\varepsilon_{\text{ij}}\right)$$

where $s_{ij}^{-}$ represents the slack variable of the i th input factor of the j th DMU; $W_{ij}$ represent the selected fiscal compensation variable; $\alpha_{i}$ denotes the corresponding estimation coefficient of fiscal compensation variable; $\varepsilon_{ij}$ denotes the error term.

Step 3 the adjustment of input-output data. By employing the fiscal compensation variables and their estimated coefficients, the fitting values of the input slack variable of each DMU are obtained:

(3) $$\hat{s_{ij}^{-}}=f\left(W_{ij},\hat{\alpha_{i}}\right)$$

The original input was adjusted following the principle of keeping the inputs of DMU which was in the worst position unchanged, while the inputs of other DMUs were increased accordingly so that all DMUs were in the identical fiscal compensation environment:

(4) $$x_{ij}^{,}=x_{ij}+\left[\mathrm{Max}\left(\hat{s_{ij}^{-}}\right)-s_{ij}^{-}\right]$$

Where $x_{ij}^{,}$ denotes the adjusted inputs; ˆ donates an estimated value; Similarly, the output value was adjusted and marked as $y_{ij}^{,}$.

Step 4 the SBM analysis of adjusted input and output. The original input-output data, $x_{ij}$ and $y_{ij}$, were replaced by the adjusted input-output values, $x_{ij}^{,}$ and $y_{ij}^{,}$respectively. Then the SBM model of the first stage was employed to calculate the management efficiency of the PV industry (ME) on which the impact of fiscal compensation had been eliminated.

According to the four-stage SBM-DEA model mentioned above, the comprehensive efficiency of the PV industry is affected by management and environmental factors. Therefore, the comprehensive efficiency (CE) of the PV industry is divided into management efficiency (ME) and environmental efficiency. Since the fiscal compensation index was chosen as the environmental variable, environmental efficiency can be expressed as the fiscal compensation efficiency (GE). The formula of fiscal compensation efficiency (GE) of ${DMU}_{d}$ in t period is as follows:

(5) $$GE_{PV}\left({DMU}_{d},t\right)=\frac{CE_{PV}\left({DMU}_{d},t\right)}{ME_{PV}\left({DMU}_{d},t\right)}$$

According to formula (5) and explanation of Dong et al., it can be seen that: if CE>ME, then GE>1, indicating that the fiscal compensation of the DMU makes up for the lack of management efficiency and improves the comprehensive efficiency; that is to say, the fiscal compensation has an incentive effect on the development of PV industry.

If CE<ME, then GE<1, indicating that the fiscal compensation of the DMU crowds out the role of management efficiency and reduces the comprehensive efficiency; that is to say, the fiscal compensation has a crowding-out effect on the development of PV industry.

If CE=ME, then GE=1, indicating that the comprehensive efficiency of the DMU is equivalent to the management efficiency, and the fiscal compensation does not change the comprehensive efficiency; that is to say, the fiscal compensation does not affect the development of PV industry.

#### 3.2.2 An analysis model of influencing factors

Based on the fiscal compensation efficiency calculated by the improved SBM-DEA four-stage model, a panel data model was employed to analyze its influencing factors:

(6) $${GE}_{at}=\alpha_{at}+\left(\sum_{i=1}^{n}\beta_{ia}X_{iat}\right)+{CTR}_{at}+\mu_{at}$$

Where a represents the enterprise; t implies the time (2010–2019); ${GE}_{at}$ is the explained variable, indicating the fiscal compensation efficiency of an enterprise in the t period; $\alpha_{at}$ is the constant term; $\beta_{ia}$ are the estimated coefficients of each explanatory variable; $X_{iat}$ is the explanatory variable, indicating the i th explanatory variable for the observation of enterprise an in the t period; n is the number of explanatory variables; ${CTR}_{at}$ denotes the control variable; $\mu_{at}$ is the random error term.

### 3.3 Variable selection

Drawing on existing research [40,50], the following variables were selected.

(1) Input and output variables. According to the theory of production function, the inputs of capital, labor, and technology are the key factors that determine the development of the industry. We selected the input index from these three input factors. In terms of capital investment, we chose net fixed assets ($x_{1}$) and main business costs ($x_{2}$); in terms of labor input, we selected the total number of employees ($x_{3}$); in terms of technology investment, we chose the total R&D expenditure ($x_{4}$). In terms of output, we chose the operating income (y) as a measure.
(2) Environment variable. The environmental variable selected was the fiscal compensation (W). Combining methods introduced by Song [51] with the information issued annually by the PV-listed companies, the government subsidy included in the current profit and loss (hereinafter referred to as government subsidy) was set as the environmental variable.
(3) Influencing factor variables. According to the hypotheses of H3 and H4, two explanatory variables are selected. The first is the fiscal compensation intensity (sub), which is measured by the ratio of government subsidies to business income; the second is the enterprise size（size）, which is measured by the natural logarithm of the total assets of enterprises
(4) Control variables. To minimize environmental interference with the model and draw on existing research [40,50,52], some characteristic variables of the enterprises were selected as the control variables. The first was the profitability (roe) which is measured by the return on net assets; the second is the equity structure (fsr) which is measured by the proportion of shares held by the first controlling shareholder; the third is the capital structure (Lev) which is measured by the asset-liability ratio; the fourth is the enterprise nature (own) which is measured by the virtual variables of the enterprise ownership nature (the value is 1 when the controlling shareholder of enterprise is national or local government, otherwise the value is 0). The fifth is the age of the enterprise (age) which is measured by the natural logarithm of the difference between the calculated year and the established year of the enterprise. Finally, the annual virtual variable (year) is added.

The variable types, symbols, and specific measurement methods are shown in Table 1.

**Table 1:** Variables, symbols and measurement.

variables	symbols	measurement	Unit
Input variable	$x_{1}$	The net value of fixed assets	Billion CNY
	$x_{2}$	Main business cost	Billion CNY
	$x_{3}$	Total number of employees	person
	$x_{4}$	Total R&D expenditure	Billion CNY
Output variable	y	operating income	Billion CNY
Environment variable	W	Government subsidies included in the profits and losses of the current period	Billion CNY
Influencing factors	Sub	Government subsidy/operating income	%
	Size	Natural logarithm of total assets	
Control variable	Roe	Return on net assets	%
	Fsr	The proportion of shares held by the first controlling shareholder	%
	Lev	asset-liability ratio	%
	Own	The value is 1 when the controlling shareholder of the enterprise is the national or local government; otherwise, the value is 0.	
	Age	Calculated year-established year, then take the natural logarithm	
	Year	The virtual variable of the year. There are 10 years and 9 virtual variables are set.	

## 4 Results and analysis

### 4.1 Calculation of fiscal compensation efficiency of China's PV industry

#### 4.1.1 Descriptive statistics of input, output, and environmental variables

For comparison, the year 2010 was taken as the base period to reduce the data of all input variables, output variables, and environmental variables. The descriptive statistics of the processed variables are shown in Table 2.

**Table 2:** Descriptive statistics of input variables, output variables and environmental variables.

variable	Average	SD	min	max	Unit
$x_{1}$	17.252	16.385	0.364	89.654	Billion CNY
$x_{2}$	41.651	35.214	1.765	175.350	Billion CNY
$x_{3}$	3268.000	6984.000	254.000	99854.000	person
$x_{4}$	2.961	1.939	0.021	10.322	Billion CNY
y	44.351	55.214	2.010	219.315	Billion CNY
W	0.290	0.404	0.026	2.057	Billion CNY

Data source: iwencai network.

Table 2 presents that there are significant variations in the resource input, output, and fiscal compensation received by PV companies in different years within the sample. This imbalance reflects the diversity of development stages, business strategies, and market environments of different companies in the industry, and provides a rich foundational dataset for analyzing the effects of fiscal compensation [53].

#### 4.1.2 Overall fiscal compensation efficiency

Table 3 presents the results of the fiscal compensation efficiency of Chinese PV listed companies calculated in this study. From the average point of view, the average fiscal compensation efficiency of front-end enterprises is 1.002, while the average fiscal compensation efficiency of back-end enterprises is 1.231. This shows that in the period of examined, the fiscal compensation efficiency of back-end enterprises is generally higher than that of front-end enterprises. The average fiscal compensation efficiency of PV-listed companies is 1.117, which is greater than 1, indicating that fiscal compensation has an incentive effect on the development of the whole PV industry. Fiscal compensation fills the deficiency of management efficiency, improves the comprehensive efficiency of the PV industry, and promotes industrial upgrading. Among the 30 PV listed companies, the average value of the fiscal compensation efficiency of 22 enterprises is greater than 1, while 8 enterprises are less than 1. It means that the listed companies with incentive effects, accounting for 73 %, are more than those with crowding-out effects. Among them, the fiscal compensation efficiency of 5 listed companies, namely, Risheng Energy, Jolywood, Jingyuntong, Eastups, and Jsjd, are all greater than 1 each year. The highest efficiency of the fiscal compensation is Solarbe, with an average annual efficiency of 2.269 and the highest annual efficiency of 2.841.

**Table 3:** Government fiscal compensation efficiency of listed PV Enterprises selected in China, 2010–2019.

Enterprise		2010	2011	2012	2013	2014	2015	2015	2017	2018	2019	mean
Front end	Flat Glass	0.983	0.854	1.098	1.002	1.011	0.899	0.925	0.972	0.986	0.996	0.973
	YiCheng Energy	1.025	0.985	1.174	1.256	1.002	1.008	0.954	0.982	1.032	0.911	1.033
	JiangNan Chemical	1.295	1.021	1.354	0.997	0.982	0.983	1.201	0.968	1.092	1.017	1.091
	JiaYu Group	0.882	0.897	1.002	0.921	1.069	0.951	0.828	0.974	0.899	0.876	0.930
	Ceepower	0.854	1.393	1.077	1.021	0.964	0.988	0.978	1.021	1.009	1.001	1.031
	Boway Group	1.080	1.359	1.291	1.284	1.131	0.889	0.820	0.932	0.826	0.800	1.041
	Golden Galss	1.036	0.836	1.180	0.868	0.921	1.062	0.854	1.104	0.804	0.885	0.955
	LinYang Energy	1.140	0.996	0.947	1.089	0.806	1.032	0.856	0.913	1.012	0.852	0.964
Back end	RiSheng Energy	1.025	0.936	1.238	1.399	1.279	1.223	1.040	1.188	1.115	0.803	1.125
	JiaWei Renewable Energy	0.831	0.959	1.005	1.221	1.231	1.105	1.075	0.981	0.922	0.713	1.004
	EGing PV	1.043	0.806	0.930	1.103	1.039	0.919	0.915	0.903	0.989	0.818	0.946
	Jolywood	1.138	1.184	0.901	1.140	1.114	1.039	1.018	1.072	1.017	0.886	1.051
	Almaden	0.867	1.505	1.485	1.541	1.591	1.447	1.326	1.120	0.863	0.704	1.245
	Ancai Hi-Tech	1.148	0.821	0.911	0.947	1.173	0.893	1.133	1.154	0.829	0.651	0.966
	Topraysolar	1.096	1.065	0.811	1.176	0.912	1.133	1.160	1.150	0.870	0.747	1.012
	JiaHua Chemical	0.840	0.908	1.484	1.454	1.341	0.875	0.813	0.925	0.887	0.895	1.042
	JingYunTong	2.984	2.162	1.761	1.819	1.865	1.937	1.923	1.744	1.678	1.505	1.938
	Sojoline	1.037	1.131	0.831	1.028	1.153	0.939	0.950	0.880	0.799	0.815	0.956
	Sunorensolar	0.944	1.094	1.171	1.100	1.021	1.176	0.837	1.150	1.098	0.804	1.040
	Sun Grow Power	1.059	1.186	0.832	1.081	0.946	0.835	0.824	1.004	0.818	0.736	0.932
	East Ups	2.279	2.129	2.077	1.978	2.188	1.924	1.936	1.846	1.962	1.503	1.982
	Akcome	0.949	0.892	1.272	1.393	1.525	1.307	0.932	0.955	0.967	0.879	1.107
	Firstpvm	0.928	0.884	1.386	1.558	1.619	1.496	1.045	0.980	0.908	0.719	1.152
	Tt-saae	0.952	0.966	0.982	1.026	1.213	1.101	1.050	1.034	1.288	0.852	1.046
	Jsjd	2.366	2.176	2.024	2.282	2.075	2.198	2.031	2.158	2.080	1.657	2.105
	Invt	1.150	0.899	1.199	0.950	0.850	1.118	1.026	1.099	0.949	0.898	1.014
	Emtco	1.189	0.943	1.024	1.179	1.234	1.440	1.199	1.057	0.898	0.868	1.103
	Solarbe	0.925	1.074	2.642	2.841	2.805	2.932	2.459	2.663	2.517	1.837	2.269
	Hnsyec	0.826	0.934	0.912	1.286	1.176	1.250	0.978	0.935	0.936	0.773	1.001
	Naura	1.065	1.097	1.203	1.365	1.143	1.150	1.079	0.930	0.765	0.722	1.052
mean	Front-end	1.037	1.043	1.140	1.055	0.986	0.976	0.927	0.983	0.958	0.917	1.002
	Back-end	1.211	1.171	1.276	1.403	1.386	1.338	1.216	1.224	1.143	0.945	1.231
	all	1.124	1.107	1.208	1.229	1.186	1.157	1.071	1.104	1.051	0.931	1.117

#### 4.1.3 Comparison of fiscal compensation efficiency between the both-end enterprises of the PV industrial chain

**Fig. 1:**
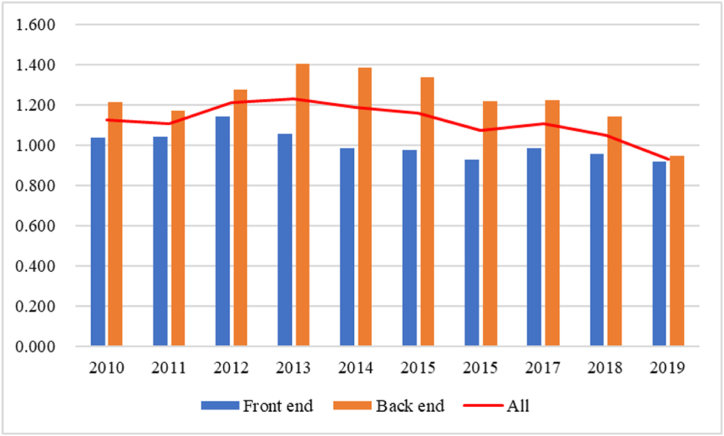
Comparison of fiscal compensation efficiency of the front-end and the back-end of the PV industrial chain and the trend of the whole industry, 2010–2019.

Using the data in Table 3, a bar chart is drawn to compare the fiscal compensation efficiency of the front- and back-end enterprises of the PV industry chain, as shown in Fig. 1.

Based on the data from Tables 3 and it is evident that fiscal compensation has a discernible incentive effect on both the front-end and back-end enterprises of the PV industry chain. The average fiscal compensation efficiency for front-end enterprises is 1.002, while for back-end enterprises it is notably higher at 1.231. This suggests that, on average, back-end enterprises are more efficient in utilizing fiscal support to enhance their operations and outputs. Furthermore, the data indicates that fiscal compensation has an incentive effect on 50 % of the 8 listed companies at the front-end of the PV industry chain and an impressive 82 % of the 22 listed companies at the back-end. This disparity in percentages further underscores the enhanced efficiency among back-end enterprises. The comparison of fiscal compensation efficiency across different years consistently shows that back-end enterprises outperform their front-end counterparts. This trend is indicative of the fact that fiscal compensation in the back-end of the PV industry yield higher returns and have a more pronounced impact. The higher fiscal compensation efficiency of back-end enterprises implies that government fiscal support is better leveraged when directed towards the latter stages of the PV industry chain. In conclusion, the analysis of fiscal compensation efficiency within the PV industry chain suggests a clear trend: fiscal support is not only an incentive for both front-end and back-end enterprises but is significantly more effective in the back-end. This insight can inform government policy and strategic planning, guiding the allocation of fiscal resources to optimize the growth and advancement of the PV industry. By focusing on the back-end, the government can promote innovation, enhance competitiveness, and drive the industry towards higher value and more sustainable development.

Therefore, hypotheses H1 and H2 are inconsistent with the experimental results. Here, we revised them as follows: fiscal compensation has an incentive effect on both-end enterprises of the PV industry chain, but the incentive effect of the fiscal compensation on the back-end is greater than that on the front-end.

#### 4.1.4 The trends in fiscal compensation efficiency

To analyze the vertical change of the fiscal compensation efficiency more intuitively with the annual growth, the trends of the fiscal compensation efficiency of the PV industry from 2010 to 2019 are also drawn in Fig. 1. It can be seen from Fig. 1 that the overall fiscal compensation efficiency of the PV industry shows a downward trend from 2010 to 2019. In 2013, the fiscal compensation efficiency of the PV industry was the highest, with an efficiency value of 1.229, indicating a significant incentive effect; In 2015 and 2019, the efficiency of the fiscal compensation decreased a lot. In 2012, the introduction of a special fiscal policy for the upgrading of the PV industry promoted the recovery of fiscal compensation efficiency. However, the policy effect lasted for a short time. In 2013, the efficiency of the fiscal compensation declined again and increased slightly in 2017, but the overall trend was downward. In 2019, the efficiency of fiscal compensation dropped to 0.931, and the incentive effect of fiscal compensation changed into crowding out effect. Therefore, the efficiency of fiscal compensation in PV industry gradually decreases over time; the incentive effect gradually decreases, while the crowding-out effect gradually increases; the introduction of fiscal policies can promote the efficiency of fiscal compensation in the short term, but it has no significant effect in the long run.

### 4.2 Analysis of the influencing factors of fiscal compensation efficiency

From the perspective of enterprise characteristics, we further analyze the factors that affect the efficiency of fiscal compensation in the PV industry to provide a policy basis for strengthening the incentive effect of fiscal compensation.

#### 4.2.1 Descriptive statistics of influencing factors and control variables

Table 4 shows that the average efficiency of the fiscal compensation is 1.117, and the standard deviation is 0.859. A great difference presents between data characteristics indicating good discreteness of the raw data. The average intensity of fiscal compensation is 1.925 %, the maximum value is 17.954 %, and the minimum value is 0.025 %, indicating that the overall fiscal compensation intensity of China's PV-listed companies is at a low level. Winsorization is performed to mitigate the impact of extreme values. Besides, after logarithmic processing, the average enterprise size of Chinese PV-listed companies is 32.954, and the standard deviation is 1.928, showing a normal distribution. The discreteness of other control variables also meets the requirements. About 66.8 % of the sample listed companies are state-owned enterprises and 33.2 % are private enterprises. The sample has a good overall representation. The variance inflation factor (VIF) is well below 5, proving that there is no multicollinearity between explanatory variables. The variation of skewness and kurtosis is relatively stable and within the standard range, which indicates that the sample data has the characteristics of concentration and small fluctuation, and has high reference and application value.

**Table 4:** Descriptive statistics of influencing factors and control variables.

Variable type	notation	Unit	Average	SD	Min	Max	VIF	kurtosis	skewness
Dependent variable	GE		1.117	0.859	0.651	2.932		−0.601	−0.128
Independent variable	Sub	%	1.925	2.016	0.025	17.954	1.702	−0.005	−0.238
	Size		32.954	1.928	15.391	26.846	1.854	−0.601	0.054
Control variable	Roe	%	9.351	16.392	−67.021	93.587	1.611	0.501	−0.223
	sr	%	42.981	14.794	7.105	101.291	2.101	−0.621	−0.221
	Lev	%	49.957	31.286	0.934	93.671	1.057	0.351	−0.305
	Own		0.669	0.415	0.000	1.000	2.016	0.606	0.030
	Age		2.964	0.327	1.390	4.218	1.920	0.551	0.227

Data source: annual reports of PV listed companies, 2010–2019.

#### 4.2.2 Estimated results

In this study, F-test which can be used to compare the advantages and disadvantages of the fixed effect model and mixed effect model and BP-LM test which can be used to test the applicability of the random effect model relative to the fixed effect model were conducted on the model setting. The results indicate that both the fixed-effect and random-effect model outperform the mixed-effect model. According to the Modified Wald test, it was found that the explanatory variables exhibit heteroscedasticity between groups. Hence, the over-identification test is utilized. The result shows that the null hypothesis of random effects is rejected. Therefore, we have ultimately selected the fixed effects model and opted for the feasible generalized least squares method to mitigate the impact of heteroscedasticity. The regression results are shown in Table 5.

**Table 5:** Regression results of factors influencing the efficiency of fiscal compensation for the PV industry.

Variable	（1）	（2）	（3）	（4）	（5）
${sub}_{-1}$		3.187*** （2.36）	8.547*** （2.08）		5.39** （2.22）
${\left({sub}_{-2}\right)}^{2}$			−32.114** （-2.20）		−21.394* （-1.18）
Size				0.291*** （6.85）	0.381*** （9.22）
Roe	−0.485** （-2.11）	−0.192 （-0.96）	−0.332 （-1.05）	0.257 （0.96）	0.628 （2.66）
Fsr	0.422*** （1.01）	0.395** （1.66）	0.385* （1.28）	0.395* （1.59）	0.584* （3.22）
Lev	−0.581** （-3.84）	−0.584*** （-2.98）	−0.332*** （-3.51）	−0.038*** （-3.11）	−0.185*** （-9.25）
Own	0.011 （0.17）	0.182 （1.04）	0.192 （1.38）	0.082 （0.96）	−0.593 （0.57）
Age	−0.194** （-3.07）	−0.124** （-1.66）	−0.157* （-1.73）	−0.117** （-3.21）	−0.392** （-1.97）
const	2.092 （8.01）	0.751 （1.32）	0.691 （1.29）	0.658* （2.39）	−0.981 （0.85）
Year	Control	Control	Control	Control	Control
WaldTest	102.33**	39.71***	42.11***	159.36***	132.82***

Note: * *, * * and * indicate that the z-test is statistically significant at 1 %, 5 % and 10 % levels, respectively.

In Table 5, column (1) is the regression of the control variable, and columns (2) and (3) are the tests for hypothesis H3.

Since the lag may exist in the impact of fiscal compensation intensity on the efficiency of fiscal compensation [54], we consider including the fiscal compensation intensity of the lag 1 period into the model for regression, as shown in column (2). The regression results show that the fiscal compensation intensity of lag 1 period has a positive impact on the efficiency of fiscal compensation at a significant level of 1 %.

To test whether there is an inverted U-shaped relationship between the intensity and the efficiency of fiscal compensation, we introduce the square term of fiscal compensation intensity (which has been centralized treatment) of lag 1 period based on column (2) regression model. The regression results demonstrated in column (3) show that the square term of fiscal compensation intensity is significantly negative, indicating that with the increase of fiscal compensation intensity, the efficiency of fiscal compensation for the PV industry is gradually improved. However, when the fiscal compensation reaches a certain degree, the efficiency of the fiscal compensation for the PV industry will gradually decrease with the increase of fiscal compensation intensity. Therefore, hypothesis H3 is verified.

Column (4) is a test of hypothesis H4. The regression results show that the enterprise size has a positive effect on the fiscal compensation efficiency of the PV industry at a significant level of 1 %. Therefore, hypothesis H4 is verified. Based on the separate regression results of the factors affecting the efficiency of fiscal compensation in the PV industry, this work puts the fiscal compensation intensity of lag 1 period and its square term into the model for regression, as shown in column (5). The results show that the impact of the first term of fiscal compensation intensity is still significantly positive, the impact of its square term is still significantly negative, and the impact of enterprise size is still significantly positive, all of which further support the results of separate regression and the verification of hypothesis H3 and H4.

From the regression results of control variables, the degree of equity concentration has a significant positive impact on the efficiency of the fiscal compensation of the PV industry. The high degree of equity concentration means that the substantial shareholder can avoid more noise interference in decision-making, and make decisions quickly, which is conducive to the efficient allocation of fiscal compensation resources, so that grass-roots management and executives can respond quickly, thus having a positive impact on the improvement of fiscal compensation efficiency. The asset-liability ratio and enterprise age have a significant negative impact on the efficiency of the fiscal compensation of the PV industry. An excessively high asset-liability ratio will increase the possibility of enterprises falling into fiscal distress. After obtaining fiscal compensation, enterprises mainly use it to repay debts and rarely spend it on, to avoid risks, investment, and R&D. Therefore, it is not conducive to the technical progress and performance improvement of enterprises, thus the efficiency of fiscal compensation is low. Enterprises established for a long time tend to have large organizations, backward management concepts and methods, slow internal operation mechanisms, rigid behavior, lack of vitality, and lack of motivation to improve production efficiency and factor structure, which is unfavorable to the improvement of fiscal compensation efficiency.

#### 4.2.3 Robustness test

In Table 5, the stepwise regression has shown that the symbols of the estimated results of each explanatory variable are consistent to a certain extent, and the significance remains consistent when the number of explanatory variables is changed. This indicates that the regression results are robust. Furthermore, drawing on relevant research [40,55], the following robustness tests have been conducted.

(1) Control endogenesis. the lagging period of fiscal compensation efficiency is introduced into the original static panel data model as an explanatory variable to construct a dynamic panel data model. The generalized method of moments is used to re-evaluate the results of the original model. The regression results are presented in Table 6.

**Table 6:** Robustness test results.

Variable	Full sample		state-owned enterprise	private enterprise
	（6）GMM	（7）（DK）	FGLS	（9）FGLS
${GE}_{-1}$	0.521*** （9.28）			
${sub}_{-1}$	5.392*** （4.95）	5.029*** （6.34）	8.354*** （6.22）	10.681** （3.24）
${\left({sub}_{-2}\right)}^{2}$	−22.395** （-2.99）	−24.951** （-6.55）	−32.001* （-5.98）	−72.151* （-3.21）
Size	0.954** （9.58）	0.399* （8.68）	0.694*** （6.21）	0.952*** （8.25）
Roe	−2.548** （-2.51）	−1.284** （-3.54）	0.857* （2.39）	0.514 （0.22）
Fsr	3.557* （3.22）	3.652*** （3.95）	0.186* （2.67）	0.671* （2.09）
Lev	−0.982*** （-3.98）	−1.35*** （-3.01）	−0.284*** （-3.98）	−0.795** （-4.01）
Own	0.364 （0.95）			
Age	−0.981*** （-6.82）	−1.528* （-3.847）	−0.394* （-2.11）	−0.514** （-4.20）
const	−8.547** （-2.67）	−2.293 （-2.51）	−8.904* （-4.92）	−4.914* （-2.57）
Year	Control	Control	Control	Control
Sargantest	0.695			
AR(1)test	0.064**			
AR(2)test	0.269			
Waldtest	309.25***		105.33***	127.93***
Fstatistics		7251.658***		
Intergroup R−squared		0.652		

Note: * *, * * and * indicate that the z-test is statistically significant at 1 %, 5 %, and 10 % levels, respectively.

The results of the validity test of the instrumental variables (Sargan test) and the correlation between the instrumental variables and the error term (Arellano-Bond test) all indicate that the instrumental variables and model settings selected in this paper are effective and appropriate. It can be seen in Table 6 that the fiscal compensation efficiency of the lagging period has a significant positive effect on the fiscal compensation efficiency of the current period, indicating that the current fiscal compensation efficiency is path-dependent on the previous fiscal compensation efficiency. The influence of the primary term of fiscal compensation intensity and firm size remains significantly positive, while the influence of the square term of fiscal compensation intensity remains significantly negative. After addressing the endogeneity issue, the results obtained from the dynamic panel data model align with those from the static panel data model. This suggests that the endogeneity problem has been successfully managed, thereby bolstering the reliability of the results.

(2) Replace the regression method. Utilizing various statistical methods or models for data analysis is a crucial aspect of robustness testing. The non-parametric covariance matrix estimation method proposed by Driscoll et al. [56] is also effective in addressing the issue of heteroscedasticity. In this study, this method is applied to regress all variables, and the regression outcomes are presented in column (7) of Table 6. The significance level and coefficients of fiscal compensation intensity, its squared term, and firm size remain consistent, indicating the robustness of the research findings from an alternative perspective.
(3) Change the sample interval. According to the nature of the enterprise, this paper divides the entire sample into two sub-samples of state-owned enterprises and private enterprises and uses the FGLS method to regress the sub-samples, respectively. The results are shown in columns (8) and (9) of Table 6. The results of grouping regression show that the significance level and symbol of fiscal compensation intensity and its square term with enterprise scale have not changed in both state-owned enterprises and private enterprises. Therefore, the conclusion is still robust.

## 5 Conclusion

Firstly, this paper theoretically analyzes the impact of fiscal compensation on industrial development. It utilizes sample data from Chinese PV listed companies from 2010 to 2019 and employs the SBM-DEA comprehensive evaluation model to calculate the fiscal compensation efficiency of sample enterprises. Furthermore, it aims to explore the heterogeneous impact of fiscal compensation on different enterprises. The main conclusions are as follows.

(1) Fiscal compensation has a positive impact on the photovoltaic industry, indicating that the government's fiscal compensation policy has effectively promoted the development of the PV industry. The average annual efficiency of fiscal compensation for China's photovoltaic industry was 1.117. This value is greater than 1, indicating that each unit of fiscal input can generate more than one unit of output. 73.3 % of the listed photovoltaic companies can significantly enhance their total factor productivity with fiscal compensation. However, for 26.7 % listed companies, fiscal compensation has a certain "crowding-out effect". This suggests that in some cases, fiscal compensation may result in suboptimal allocation of resources. The overall efficiency of fiscal compensation shows a fluctuating downward trend, which may indicate that the marginal benefits of fiscal compensation are decreasing, or the industry is facing increasing challenges and problems.
(2) The incentive effect of fiscal compensation is greater on the back end of the photovoltaic industry chain than on the front end. The average efficiency of fiscal compensation at the front end of the photovoltaic industry chain is 1.002, and it has an incentive effect on 50 % of enterprises. At the back end, the average efficiency of fiscal compensation is 1.231, and it has an incentive effect on 82 % of enterprises. The results above demonstrate a significant disparity in the incentive coverage of fiscal compensation between front-end and back-end enterprises in the photovoltaic industry chain.
(3) Different fiscal compensation strategies may have varying effects. There is an inverted U-shaped relationship between the intensity of fiscal compensation and the efficiency of fiscal compensation in the photovoltaic industry. This implies that there is an optimal level of fiscal compensation for maximizing efficiency in the industry. Enterprises with larger scale and higher equity concentration may be able to make more effective use of fiscal compensation to improve efficiency due to their richer resources, more mature management, fewer agency problems, and other factors. Enterprises with a higher asset-liability ratio and older age may experience a negative impact on the efficiency of fiscal compensation due to increased fiscal risks and a more rigid management structure.
(4) This study adopts a comprehensive framework for assessing policy efficiency, explores influencing factors, and empirically examines China's photovoltaic industry as an example. However, due to space limitations, there has been no in-depth study on the mechanisms through which influencing factors exert their influence. Future studies will address these deficiencies.

## 6 Policy implications

Based on the above conclusion, fiscal compensation is a feasible way to improve the total factor productivity, promote industrial upgrading, and achieve high-quality development of China's PV industry. However, the "incentive effect" of fiscal compensation on the development of the PV industry should be further strengthened, and efforts should be made from the following aspects to change the increasingly prominent trend of crowding out effect:

Firstly, optimize the fiscal compensation structure. Formulate differentiated policies based on the scale, development stage, and specific needs of the enterprise. For enterprises that have reached a mature stage and possess strong market competitiveness, it is advisable to gradually decrease direct fiscal subsidies and instead promote self-development through market mechanisms. When formulating policies, it is important to thoroughly consider the marginal effects of fiscal compensation to prevent any potential crowding out effects on enterprises while maintain the incentive effect using scientific methods.

Secondly, reasonably adjust the distribution ratio of fiscal compensation based on the actual needs and incentive effects of front-end and back-end enterprises. Priority will be given to supporting back-end links such as technology research and development, updating production equipment, and market development to enhance the added value of the entire industrial chain. Encourage front-end and back-end enterprises to strengthen cooperation and create synergy within the industrial chain. The government can promote technology and resource sharing between front- and back-end enterprises by providing subsidies for cooperative projects and establishing industrial alliances.

Thirdly, optimize fiscal compensation intensity. Based on the inverted U-shaped relationship, determine the optimal intensity level of fiscal compensation to prevent excessively high or low fiscal compensation. The government should determine the most suitable compensation level for the PV industry through pilot projects and data analysis to maximize compensation efficiency. Regularly evaluate the impact of fiscal compensation, and adjust the compensation intensity dynamically based on industry development and market changes to keep it at the optimal level.

## Data availability statement

Publicly available datasets were analyzed in this study. Data sets used or analyzed in the current study are available from corresponding authors upon reasonable request.

## CRediT authorship contribution statement

**Jing Li:** Writing – review & editing, Writing – original draft, Visualization, Validation, Supervision, Software, Resources, Project administration, Methodology, Investigation, Formal analysis, Data curation, Conceptualization.

## Declaration of competing interest

The authors declare that they have no known competing financial interests or personal relationships that could have appeared to influence the work reported in this paper.
